# Potential Role of Probiotics for Inflammaging: A Narrative Review

**DOI:** 10.3390/nu13092919

**Published:** 2021-08-24

**Authors:** Nikolina Jukic Peladic, Giuseppina Dell’Aquila, Barbara Carrieri, Marcello Maggio, Antonio Cherubini, Paolo Orlandoni

**Affiliations:** 1 Clinical Nutrition, IRCCS INRCA Ancona, 60127 Ancona, Italy; n.jukicpeladic@inrca.it (N.J.P.); p.orlandoni@inrca.it (P.O.); 2 Geriatria, Accettazione Geriatrica e Centro di Ricerca per L’invecchiamento, IRCCS INRCA, 60127 Ancona, Italy; carrieribarbara@gmail.com (B.C.); a.cherubini@inrca.it (A.C.); 3 Department of Medicine and Surgery, University Medical School of Parma, 43121 Parma, Italy; marcellogiuseppe.maggio@unipr.it; 4 Geriatric Clinic Unit, University-Hospital of Parma, 43121 Parma, Italy

**Keywords:** inflammaging, probiotics, aging, healthy older subjects

## Abstract

Background and aims: Inflammaging, a chronic, low-grade inflammation (LGI), is one of the mechanisms of adaptation of an organism to aging. Alterations in the composition of gut microbiota and gut permeability are among the main sources of LGI. They may be modulated by supplementation with live microorganisms, i.e. probiotics. This narrative review was performed with the aim to critically examine the current evidence from randomized clinical trials (RCTs) on the effects of probiotics on pro-inflammatory and anti-inflammatory cytokines and C-reactive protein (CRP) in healthy older subjects. Methodology: RCTs on the effects of probiotics on inflammatory parameters in subjects older than 65 years published in English and Italian from 1990 to October 2020 were searched in PubMed. Studies that were not RCTs, those using probiotics together with prebiotics (synbiotics), and studies performed in subjects with acute or chronic diseases were excluded. The findings of RCTs were reported in accordance with the Preferred Reporting Items for Systematic Reviews and Meta-Analyses (PRISMA). Results: A total of nine RCTs met the eligibility criteria and were included in this narrative review. Four articles reported that probiotic supplementation significantly affected inflammatory parameters, respectively, by reducing TGF-β1 concentrations, IL-8, increasing IL-5 and Il-10, and IFN-γ and IL-12. Conclusions: Based on this narrative review, probiotic supplementation showed a limited effect on inflammatory markers in healthy individuals older than 65 years. Besides being few, the studies analyzed have methodological limitations, are heterogeneous, and provide results which are incomparable.

## 1. Introduction

Independent of variations in fertility, mortality, and migrations, the number of older subjects in the world will continue to increase [1]. Global population aging is the main demographic phenomenon of the twenty-first century, and it is going to have a profound impact on our societies. Therefore, achieving a more thorough understanding of aging and age-related chronic diseases has become the main objective of gerontological and geriatric research. According to accumulating scientific data, aging and age-related diseases share some common biological mechanisms [2,3]. One of the most relevant processes is chronic, low-grade inflammation (LGI), the so-called inflammaging. Pro-inflammatory factors increase in older subjects as a consequence of prolonged stimulation of innate immune system by different agents and of a progressive increase in senescent cells that produce inflammatory molecules [4]. Studies performed on centenarians found that in these long-lived subjects, the constant alert state in which the immune cells are kept by low-grade inflammation is also present, but it is counterbalanced by an effective anti-inflammatory response (anti-inflammaging) [5,6,7]. The imbalance between pro- and anti-inflammatory factors is one of mechanisms at the basis of several age-associated diseases such as type 2 diabetes, Alzheimer’s disease, Parkinson’s disease, cardiovascular diseases, osteoarthritis, sarcopenia, major depression, and many types of cancer [8,9,10,11]. On these premises, current research is pursuing the identification of factors that could modulate inflammation by acting on its sources, among which there are also alterations in the composition of gut microbiota and gut permeability [12]. Aging, diet, and pharmacotherapy can cause the imbalance of the structure and function of the gut intestinal microbial communities (dysbiosis), leading to increased gut permeability and a higher translocation of substances from gut lumen into the circulation, thus increasing chronic low-grade inflammation [13,14,15,16,17,18]. The improvement in gut microbiota composition by supplementation with live microorganisms—probiotics—has been advocated as a promising strategy to ameliorate dysbiosis [19,20]. It has been found that, when administered in adequate amounts, probiotics may enhance and/or modulate the functionality of existing microbial communities, influence systemic and mucosal immune function, and improve intestinal barrier function [21,22,23,24]. In a recent review, Mohr et al. evaluated the effect of probiotic supplementation on circulating immune and inflammatory markers in healthy adults (aged 18–65 years). The authors concluded that probiotics had a limited effect on immune and inflammatory markers [25]. The aim of the present narrative review was to critically examine the current evidence from randomized clinical trials concerning the ability of various probiotics to affect pro-inflammatory and anti-inflammatory cytokines as well as C-reactive protein (CRP) in healthy older subjects (aged older than 65 years).

## 2. Materials and Methods

A literature search was performed with the aim to identify and retrieve randomized clinical trials (RCTs) on the effects of probiotics on inflammatory parameters in older subjects (age older than 65 years). Studies that were not RCTs, those using probiotics together with prebiotics (symbiotics), and studies performed in subjects with acute or chronic diseases were excluded. The authors searched in PubMed studies published from 1990 to October 2020. Only articles published in the English and Italian languages were considered. The words used in the search were different combinations of the following terms: “probiotics”, “probiotic supplements”, “low grade inflammation”, “systemic immunity”, “immunosenescence”, “immunomodulation”, “immune response”, “immune function”, “cytokines”, “older”, “elderly”, and “geriatrics”. Reference lists of all included articles and of recent reviews and meta-analyses were searched for additional literature [26,27,28]. Two authors (NJP and GD) independently evaluated the list of articles and selected the most relevant of them, excluding duplicates. The senior authors (AC and PO) resolved disagreements. A total of 9 RCTs on the influence of probiotics on LGI were identified and analyzed by the authors. The characteristics and main results of each trial are summarized in Table 1. In the absence of specific guidelines for the presentation of the results of the narrative reviews, reporting of findings was conducted, as much as possible, in accordance with the Preferred Reporting Items for Systematic Reviews and Meta-Analyses (PRISMA) [29].

## 3. Results

Overall, nine randomized controlled trials assessing the efficacy of supplementation with probiotics on modulating the inflammatory parameters in healthy older subjects were identified. The studies identified were extremely heterogeneous with respect to study settings, methodologies used, and bacterial strains administered; thus, the main elements and findings of each study are presented in the text below separately, while the principal characteristics of each study are summarized in Table 1.

**Table 1:** Randomized controlled trials assessing the efficacy of supplementation with probiotics on modulating inflammatory parameters.

Reference	Study Sample (Age, n. of Subjects Enrolled)	Inclusion Criteria	Probiotic and Placebo Characteristics and Dosage	Duration of Administration and Follow Up	Effect on Inflammatory Markers
De Simone et al. (1992)	Institutionalized older. Mean age 76 years; *n* = 25 subjects enrolled (*n* = 15 intervention vs. *n* = 10 control).	Written informed consent from participants, older than 70 years, no overt diseases according to anamnesis and no fever, pain, cough, dysuria, modification of bowel habits etc.	2 capsules × 4 times/day, containing combined B. bifidum (10^9^ CFU) and L. acidophilus (10^9^ CFU) vs. 2 capsules × 4 times a day of placebo, containing saccarose and gelatin	4-week intervention	No effect on plasma TNF-α
Guillemart et al. (2010)	Free-living older. Mean age 76 years (range 69–95); *n* = 1072 subjects enrolled in the study.	Both gender, ≥70 years, free living, AGGIR score between 5 and 6, vaccination ag. influenza virus at least 14d before inclusion, MMSA score ≥24, BMI between 17 and 25 kg/m^2^, compliance with a dietary restriction (no fermented dairy products with other probiotics, yoghurts and medication containing probiotics, vitamins, minerals and other nutrients) during 2 previous weeks and throughout the study, written informed consent.	2 bottles, 100 g each/day, of fermented diary drink containing at least 10^10^ CFU/100 g of the probiotic strain L. Casei DN-114001 vs. non fermented diary drink	12-week intervention + 4-week follow up	No effect on blood CRP, IL-1, IL-6, IFNα, IFNβ, IFNγ, IL-8, IL-10, IL-12 or TNF-α < βγ
Mañe et al. (2011)	Institutionalized older. Mean age 70 years (range 65–84); *n* = 60 subjects enrolled; *n* = 20 placebo, *n* = 20 low dose probiotic, *n* = 20 high dose probiotic.	Written informed consent, older than 65 years.	20 g of powdered skilled milk containing 5 × 10^8^ CFU/day of *L. plantarum* CECT7315/7316 (low probiotic dose) or 5 × 10^9^ CFU/day of *L. plantarum* CECT7315/7316 (high probiotic dose) or 20 g of powdered skilled milk (placebo)	12-week intervention + 12 week follow up	TGF-β decreased (value not given) independent from probiotic dosage
Moro-García et al. (2013)	Free-living older. Mean age 70 years (range 65–90); *n* = 61 subjects enrolled.	Older than 65 years, treatment in determined Spanish health centers, written informed consent.	3 capsules/day containing at least 3 × 10^7^ *L. delbrueckii* subs bulgaricus 8481 vs. placebo capsules with corn starch	24-week intervention	Plasma IL-8 decreased (value not given), hBD-2 increased (value not given); no effect on IFN-γ, IL- 1β, IL-2, IL-4, IL-5, IL-6, IL-10, IL-12p70, TNF-α
Dong et al. (2013)	Free-living older. Range 55–74 years; *n* = 30 subjects enrolled; *n* = 16 intervention group vs. *n* = 14 placebo.	Age 55–80 years, BMI 19–30 kg/m^2^, good general health, written informed consent.	2 × 65 mL/day probiotic drink containing 6.5 × 10^9^ CFU/bottle *L. casei* Shirota vs. 130 mL of skimmed milk/day	4-week intervention + 4 weeks of washout	No effect on blood CRP, IL-10/Il-12 ratio increased for LPS- stimulated PBMC
Valentini et al. (2015)	Free living healthy older. Mean age 70.1 ± 3.9 years; *n* = 69 enrolled (*n* = 35 intervention vs. *n* = 34 controls).	Healthy individuals aged 65–85 years, BMI 22–30 kg/m^2^ and Eastern Cooperative Oncology Group Performance Status (ECOG) 0–2, able to use a computer and with access to the internet, by themselves or with help.	RISTOMED personalized diet and 2 capsules/day containing 112 billion lyophilized bacteria consisting of *B. infantis* DSM 24737, *B. longum* DSM 24736, *B. breve* DSM 24732, *L. acidophilus* DSM 24735, *L. delbrückii* ssp. bulgaricus DSM 24734, *L. paracasei* DSM 24733, *L. plantarum* DSM 24730, and *S. thermophilus* DSM 24731 vs. RISTOMED personalized diet	8-week intervention (56 ± 2 days)	No effect on hsCRP
Nyangale et al. (2015)	Free-living older 65–80 years, *n* = 17 subjects probiotic period 1, placebo period 2, *n* = 17 subjects placebo period 1, probiotic period 2.	Age 65–80 years, written informed consent.	1 capsule/day containing 10^9^ CFU of Bacillus coagulans GBI-36, 6086 (BC30) per day vs. capsules containing microcrystalline cellulose	2 treatment periods consisting of 4-week intervention separated by 3-week washout period	No effect on IL-10, TNF-α or CRP
Spaiser et al. (2015)	Free-living healthy older, range 65–80 years; *n* = 42 subjects enrolled.	Written informed consent	2 capsules/day containing a powder mixture of *L. gasseri* KS-13, *B. bifidum* G9-1, *B. longum* MM2 for a total of 3 × 10^9^ viable cells / day vs. capsules containing potato starch and silicon dioxide	3-week intervention and 1-week post intervention for each period of crossover + 5-week washout between the intervention periods	IFN-γ increased after period 1 in intervention and after period 2 in both groups, IFN-γ increased after period 2 in both groups, IL-5 and IL-10 increased with probiotic interventions during both periods
Lee et al. (2017)	Free-living older. Mean age placebo 65.7 ± 0.56 years, probiotic, 65.7 ± 0.50 years; *n* = 200 subjects enrolled.	Non diabetic (fasting serum glucose concentration < 126 mg/dL), age > 60 years, white blood cell levels between 4 × 103/µL and 10 × 103/µL, written informed consent.	1 bottle (120 mL)/day of yogurt containing *L. paracasei* (*L. casei* 431^®^) at 12.0 × 10^8^ CFU/day, *B. lactis* (BB-12^®^) at 12.0 × 10^8^ CFU/day and 0.0175% heat-treated *L. plantarum* (nF1) per day vs. 120 mL of milk	12-week intervention	IL-12 and IFN-γ increased, no effect on CRP

CFU, colony forming unit; TNF, tumor necrosis factor; CRP, C-reactive protein; hCRP, high sensitivity C-reactive protein; IL, interleukin; IFN, interferon; TGF, transforming growth factor; hBD-1, human beta defensin; C5a, complement factor 5a, LPS, lipolysaccharide; PBMC, peripheral blood mononuclear cell.

The first study meeting the inclusion criteria for this review dates back to the year 1992. In that year, De Simone et al. performed a randomized controlled trial to investigate the effect of supplementation of *Bifidobacterium bifidum* (BB) and *Lactobacillus acidophilus* (LA), contained in capsules of a specific, commercially available product, on the immune system in a group of older volunteers with no overt diseases [30]. The study was carried out on a small sample of subjects whose blood values were perfectly comparable at baseline. Fifteen subjects were assigned to the intervention group (mean age 76 ± 8 years), ten to the control group (mean age 75 ± 11 years). The authors found that a 4 week period treatment with BB and LA was not sufficient to affect TNF-α levels, which moved from 1.33 ± 5.1 pg/mL at T1 (enrollment) to 1.5 ± 5.1 pg/mg at T2 (*p* > 0.05) in the intervention group, and did not change at all in the placebo group (0 pg/mL both at T1 and T2). Almost 20 years later another group of authors led by Guillemard performed a multi-centric, double blind, controlled, parallel follow-up study in 1072 free living older volunteers to assess whether the consumption of a fermented dairy product containing *Lactobacillus casei* DN-114001 may affect the resistance of the older to common infection diseases [31]. Subjects from the intention to treat population, whose baseline characteristics were well balanced, were randomly assigned to consume 200 g/day of a sweetened, flavored fermented diary product containing at least 10^10^ CFU/100 g of *L. casei* DN-114001 (intervention) or the same quantity of placebo (sweetened, flavored, non-fermented diary product), for a consistently longer period than in the study by De Simone et al. After the three-month intervention, the authors performed blood examinations in a subpopulation of 125 subjects who were randomly selected from the overall sample (63 randomized to fermented product, 62 to control) to assess the changes in biological and immunological parameters following the intake of *L. casei* DN-114001. They tested numerous biological and immunological parameters—CRP, IL-1, IL-6, IFNα, IFNβ, IFNγ, TNF-α, IL-12, IL-10, and IL-8—but, despite a quite long period of administration, they found that none of the parameters tested were modified. A study with similar objectives was carried out a year later by Mañe et al. (2011). The authors investigated the effects of the administration of probiotics *Lactobacillus plantarum*, CECT 7315 and CECT 7316, mixed at a 1:1 ratio, on the systemic immunity in 60 institutionalized healthy older subjects. The parameters tested were TGF-β1, IL-1, and IL-10 [32]. In their study, the authors randomly assigned 20 subjects to high probiotic dose mixture (5 × 10^9^ CFU/day), 20 subjects to low probiotic dose mixture (5 × 10^8^ CFU/day), and 20 subjects to a placebo group. The three groups were perfectly comparable at baseline for their demographic and nutritional characteristics and for values of all routine laboratory parameters. Per protocol analyses was performed to assess the effect of supplementation on systemic inflammation after 12 weeks of administration and after 24 weeks (12 weeks of administration and 12 weeks follow up). The plasma concentrations of IL-1 and IL-10 were undetectable at every time point, but in this study, the authors found that the values of TGF-β1 concentrations were significantly affected and reduced after the administration of probiotics in both the low-dose and high-dose probiotic groups compared to placebo (*p* < 0.05 after 12 weeks, *p* < 0.01 after 24 weeks).

In 2013, Moro-Garcia et al. analyzed the effect of supplementation with *Lactobacillus delbrueckii supsp. bulgaricus* 8481 on the innate and acquired immune response of older subjects tested in 2013 in [33]. Within a multi-centric, double-blind, placebo-controlled study, twenty-eight subjects who were assigned to the intervention group consumed three capsules/day containing 3 × 10^7^ *L. delbruecki supsp. bulgaricus* 8481 for 6 months, twenty-four subjects consumed a placebo. Subjects from the two groups were perfectly comparable at baseline relatively to demographic data and hematological, biochemical, and immunological values. The authors tested numerous cytokines with pro and anti-inflammatory activities, both at 3 and at 6 months - IFN-γ, IL- 1β, IL-2, IL-4, IL-5, IL-6, IL-10, IL-12p70, TNF-α, and TNF-β - and found that the 6 month consumption of *L. delbruecki supsp. bulgaricus* 8481 affected only IL-8 levels, which were significantly reduced in the probiotic group. In the same year, Dong et al. performed a randomized placebo-controlled, single-blind crossover study in a small sample of 30 healthy older volunteers (55–74 years old) to investigate the effect of *Lactobacillus casei Shirota* (LcS) contained in a commercial fermented probiotic drink on their immune function [34]. Subjects were randomized to enter two intervention arms—probiotic (16 subjects) and placebo (14 subjects)—and during the 4 weeks of the intervention period consumed two bottles of the product or placebo daily. After a 4 week post-administration washout period, subjects were crossed over to the other treatment. The effect of product on inflammation was assessed by measuring CRP and C5a markers, IL-10 and Il-12. The only statistical significance was registered relatively to the marginal increase in the ratio of IL-10/Il-12 during the period of treatment with probiotics compared to placebo treatment. In 2015, Valentini et al. compared the changes in high-sensitivity C-reactive protein (hsCRP) levels in 31 subjects who were consuming the personalized diet (Arm A) and 31 subjects who were consuming personalized diet and a probiotic supplement (Arm B) within a multicenter open label, randomized, controlled trial: the RISTOMED project [35]. The probiotic used contained 112 × 10^9^ of lyophilized bacteria *Bifidobacterium infantis* DSM 24737, *B. longum* DSM 24736, *B. breve* DSM 24732, *Lactobacillus acidophilus* DSM 24735, *L. delbrückii* ssp. *Bulgaricus* DSM 24734, *L. paracasei* DSM 24733, *L. plantarum* DSM 24730, and *Streptococcus thermophilus* DSM 24731. The authors enrolled subjects whose baseline values of hsCRP were slightly above the normal range (≥3 mg/L), suggesting some level of low-grade inflammation (68% of subjects from Arm A and 71% of subjects from Arm B, without significant differences between the two groups), and found that the eight-week consumption of probiotics was not efficient in modulating the values of inflammatory parameters. In the same year, Nyangale et al. performed a randomized, double-blind, placebo-controlled crossover study to test the efficacy of a commercially available spore-forming probiotic capsule containing 10^9^ CFUs of *Bacillus coagulans* GBI-36, 6086 (BC30) in improving immune and gut function in healthy older subjects [36]. Forty-two volunteers aged 65–80 years, free from chronic diseases, were randomly allocated into group A (intervention) or B (placebo). The study contained two treatment periods consisting of 4 weeks separated by a 3 week washout period. For the first 4 weeks, subjects from group A consumed BC30 and subjects from group B consumed the placebo (microcrystalline cellulose). After the washout period, the products were inverted. Samples of feces and blood were analyzed at the beginning of each treatment (probiotic and/or placebo) and after the 4 week treatment to assess the comparative effects. Parameters tested were IL-10, TNF-α, and CRP but neither Nyangale et al. found any significant difference in the values between the two groups after the administration of *Bacillus coagulans* GBI-36, 6086 (BC30). Spaiser et al. also performed in the same year a 13 week randomized, double-blind, placebo-controlled crossover study in a small sample of healthy older adults (mean age 70 + 1 years) to assess the effect of a specific probiotic mixture of *Lactobacillus gasseri* KS-13, *Bifidobacterium bifidum* G9-1, and *Bifidobacterium longum* MM2 on circulating CD4+ lymphocytes, cytokine production, and intestinal microbiota [37]. Thirty-four participants were randomly assigned to one of two intervention sequences. All participants completed a one-week pre-intervention phase followed by a 3 week intervention and a one-week post-intervention for each period of the crossover, with 5 weeks of washout between the intervention periods. To evaluate the effect of probiotics on inflammation, cytokine concentrations were assessed at baseline and after the first and second intervention. Spaiser et al. identified some important changes in the values of parameters tested. IFN-γ increased after probiotic intervention versus placebo in period 1 and that difference was maintained during the washout period. During period 2, IFN-γ production increased significantly with both interventions (*p* < 0.0001) without differences between them. IL-2 increased with both interventions in the period 2, IL-5 and Il-10 also increased, but only with probiotic interventions during both periods. Finally, the most recent study assessing the efficacy of supplementation with probiotics on modulating the inflammatory parameters in healthy older subjects dates back to the year 2017. In that year, Lee et al. conducted an open-label, placebo-controlled study to investigate the impact of the consumption of yogurt containing *Lactobacillus paracasei* ssp. *paracasei* (*L. paracasei*), *Bifidobacterium animalis* ssp. *lactis* (*B. lactis*) and heat-treated *Lactobacillus plantarum* (*L. plantarum*), on immune function in a large sample of healthy volunteers older than 60 years [38]. Volunteers were randomly assigned to the intervention group (100 subjects) which consumed one bottle (120 mL) of dairy yogurt a day, containing probiotics, or to the placebo group (100 subjects) which consumed the same volume of milk (placebo), once a day. TNF-α, IL-12, IFN-γ, and high sensitivity C-reactive protein (hsCRP) were perfectly comparable at baseline between the two groups. After 12 weeks of treatment, the intervention group registered an increase in IFN-γ concentrations compared to placebo (*p* < 0.041) and in IL-12 (*p* < 0.01). HsCRP values registered a statistically significant increase in the placebo group (from 0.80 mg/L ± 0.007 at t0 to 2.01 mg/L ± 0.71 after 12 weeks; *p* < 0.05) and did not change in the intervention group (1.24 ± 0.26 vs. 1.77 ± 0.50, *p* > 0.05).

## 4. Discussion

The results of some studies on healthy centenarians suggest that the formula of longevity lies in the balancing low-grade inflammation, which is the basis of a large number of age-related diseases, with anti-inflammatory factors. Evidence is also available on capability of microorganisms contained in probiotics in treating the causes of dysbiosis, which is one of the main causes that increases chronic low-grade inflammation [39]. In this study, we searched human RCTs investigating the impact of probiotics on inflammation by assessing the values of biomarkers of inflammation, i.e., cytokines and C-reactive protein. The results of our research show that, despite the high expectations for probiotics, the clinical trials carried out with the aim of analyzing their effect on inflammation were few, extremely heterogeneous, and provided conflicting results. Therefore, the available evidence is not sufficient to support the concept that probiotics might be a useful tool to counteract inflammaging in healthy older adults. The main reasons underlying this inconclusiveness of published research might be related, at least in part, to the methodological limitations and heterogeneity of the different studies. Concerning the first point, the majority of trials were performed in small samples, with the only exception of Guillemart et al. (*n* = 125 subjects) and Lee et al. (*n* = 152). The inclusion of older people in clinical trials may actually be challenging given that older subjects may have cognitive impairment, which prevents them from being able to consent to participate in the trial, a higher likelihood of becoming sick, or sensory or mobility limitations that reduce their ability to participate without the help of a family member or caregiver [40,41]. Still, the sample dimensions are of great importance for generalization of study results. Secondly, the studies were characterized by short follow-up periods. Given that there are no clear indications by the scientific community on what would be the right duration of administration of probiotics, it seems that the administration periods were defined almost arbitrarily within each study. They went from 4 up to 24 weeks. Moreover, a number of inaccuracies in study design and statistical analysis may be highlighted that also limit the generalizability and reproducibility of their results. In most studies the recruitment, randomization, and allocation concealment are poorly described. Protocols used to guarantee the adherence and compliance are also rarely specified while that would be extremely important, especially for those studies where products were consumed several times a day and/or for a very long time. For data analyses, the final, per protocol analyses was prevalently used, while the intention to treat is a gold standard for RCTs. RCTs analyzed are also heterogeneous relatively to outcome measures assessed, probiotic strains, and doses administered. Given that the authors assessed the effects of probiotics on variables which differed from study to study—different pro- and anti-inflammatory cytokines, tumor necrosis factor and transforming growth factor, CRP, or hsCRP—even the results of studies that found probiotics effective in modulating the LGI are incomparable. Mane et al. reported on the effect of probiotics on TGF-β, Moro-Garcia on IL-8, Dong et al. on the IL-10/IL-12 ratio, Spaiser et al. on IL-5 and IL-10, and Lee et al. on IL-12 and IFN-γ. In addition, it has to be considered that different laboratories use different reagents and measurement techniques, that have different levels of accuracy and are not always adequate to answer the study questions. This is the case, for instance for CRP which, if measured with methods other than high-sensitivity ones, cannot be used to demonstrate a decrease at a value lower than 0.5 g/L. Microorganisms administered in different studies were mostly from the *Lactobacillus* and *Bifidobacterium* genera, but species and strains were quite different just like the combinations of microorganisms for each product. Furthermore, in some studies, probiotics were administered added to dairy products—fermented and non-fermented, which naturally contain probiotics, and given that the health effect is carried by the entire product and not only by bacterial strains, the results of those studies may be affected by this consideration. Just like the duration of the administration period, the dosage of probiotic bacterium administered daily varied consistently among the studies (from 10^7^ to 10^10^ CFU). The definition of a proper dosage is a problem of primary importance in this field given that it is not clearly defined by the scientific evidence and/or by the relevant institutions [42]. Very few countries have regulations on probiotics, which differ consistently among each other. The Italian Ministry of Health, for example, developed the guidelines on probiotics and prebiotics in 2018 and according to those guidelines, probiotics must contain no less than 10^9^ live cells of at least one strain [43]. The evidence collected through in vitro studies and studies on rats and humans regarding the functioning of the microbiota and the ability of probiotics, in particular those containing Lactobacillus and Bifidobacterium species, to modulate its composition, is available and is very promising. The ability of probiotics to affect positively different pathologies in different age groups was also tested and partially proved, but the evidence collected so far on the efficacy of probiotics in modulating LGI in older subjects is poor and inconsistent. As already pointed out, performing studies in this population is particularly difficult and challenging but considering the importance of the topic and some positive results found in other age groups, research in this area should continue. Future studies should be performed in significantly larger samples, with study designs which will overcome the weaknesses evidenced in this review. Within those studies it would be particularly useful to collect additional evidence on how the modulation of biomarkers of inflammation following the intake of probiotics is associated with clinical outcomes. Finally, when interpreting the results of studies, it is very important to keep in mind that immunosenescence and inflammaging, which until recently were considered exclusively negative factors, in reality are a result of physiological remodeling during aging. It has been shown, indeed, that in centenarians the increased inflammatory state does not have negative effects on the organism, as it is balanced by the production of anti-inflammatory substances [44].

## 5. Conclusions

Based on this narrative review, probiotic supplementation showed a limited effect on inflammatory markers in healthy subjects older than 65 years. Besides being few, the studies analyzed have methodological limitations, were heterogeneous, and provided results that are incomparable.

## Data Availability

The data presented in this study are available on request from the corresponding author.
